# A 3′-Untranslated Region (3′UTR) Induces Organ Adhesion by Regulating miR-199a* Functions

**DOI:** 10.1371/journal.pone.0004527

**Published:** 2009-02-18

**Authors:** Daniel Y. Lee, Tatiana Shatseva, Zina Jeyapalan, William W. Du, Zhaoqun Deng, Burton B. Yang

**Affiliations:** 1 Sunnybrook Research Institute, Sunnybrook Health Sciences Centre, Toronto, Canada; 2 Department of Laboratory Medicine and Pathobiology, University of Toronto, Toronto, Canada; Lehigh University, United States of America

## Abstract

Mature microRNAs (miRNAs) are single-stranded RNAs of 18–24 nucleotides that repress post-transcriptional gene expression. However, it is unknown whether the functions of mature miRNAs can be regulated. Here we report that expression of versican 3′UTR induces organ adhesion in transgenic mice by modulating miR-199a* activities. The study was initiated by the hypothesis that the non-coding 3′UTR plays a role in the regulation of miRNA function. Transgenic mice expressing a construct harboring the 3′UTR of versican exhibits the adhesion of organs. Computational analysis indicated that a large number of microRNAs could bind to this fragment potentially including miR-199a*. Expression of versican and fibronectin, two targets of miR-199a*, are up-regulated in transgenic mice, suggesting that the 3′UTR binds and modulates miR-199a* activities, freeing mRNAs of versican and fibronectin from being repressed by miR-199a*. Confirmation of the binding was performed by PCR using mature miR-199a* as a primer and the targeting was performed by luciferase assays. Enhanced adhesion by expression of the 3′UTR was confirmed by *in vitro* assays. Our results demonstrated that upon arrival in cytoplasm, miRNA activities can be modulated locally by the 3′UTR. Our assay may be developed as sophisticated approaches for studying the mutual regulation of miRNAs and mRNAs *in vitro* and *in vivo*. We anticipate that expression of the 3′UTR may be an approach in the development of gene therapy.

## Introduction

Human Genome Project identified approximately 25,000 protein-coding genes, occupying 1.9% of total genomic DNA. The remaining DNA has come to be known as “junk” DNA or cellular detritus and was presumed to serve no particular function because it does not code proteins. Pseudogenes are part of these non-functional DNAs since they are the defective copies of the protein-coding genes. In fact, pseudogenes are nearly as abundant as the protein-coding genes and therefore appear to be an important component of the genome. It has been reported that there are approximately 20,000 putative pseudogenes in the human genome [1], [2]. A large number of pseudogenes are found to be transcribed. Analysis of chromosome 22 indicated that approximately 20% of the pseudogenes are potentially transcribed [3]. Pseudogene transcription has also been reported in other species including fly, mouse, cow, and chimp [4]. The assumption that pseudogenes are dysfunctional is based on the fact that pseudogenes do not code for proteins. However, it is unknown whether non-coding RNAs can affect microRNAs (miRNAs) functions.

miRNAs are single-stranded RNA of 18–24 nucleotides in length and are generated by an RNase III-type enzyme from an endogenous transcript that contains a local hairpin structure [5], [6]. miRNA functions as a guide molecule in post-transcriptional gene silencing by partially complementing with the 3′-untranslated region (3′UTR) of the target mRNAs, leading to translational repression [7]–[9]. By silencing various target mRNAs, miRNAs have key roles in diverse regulatory pathways, including controlling development [10]–[15], cell differentiation [16]–[20], apoptosis [21], [22], cell proliferation [23]–[25], division [26], [27], protein secretion [28], [29], immuno-response [30], viral infection [31]–[34], and cancer development [35]–[47]. Functionally, miRNAs are opposite to transcription factors, which turn on genes, while miRNAs function as modulators by down-regulating gene expression [7], [48]. Studies associated with miRNA functions and regulations of gene expression by mature miRNAs are overwhelming. However, it is unknown whether the mature miRNAs can also be regulated. We hypothesize that the mRNA 3′UTRs can bind to and regulate miRNA functions. In this study, we used versican 3′UTR as a model to study the role of the non-coding transcript. Versican is an extracellular proteoglycan that has been shown to mediated varieties of cell activities [49]–[53]. We found that the expression of the versican 3′UTR induced cell, tissue, and organ adhesion by arresting miR-199a* functions.

## Results and Discussion

### Transgenic expression of versican 3′UTR induces organ adhesion

**Figure 1:**
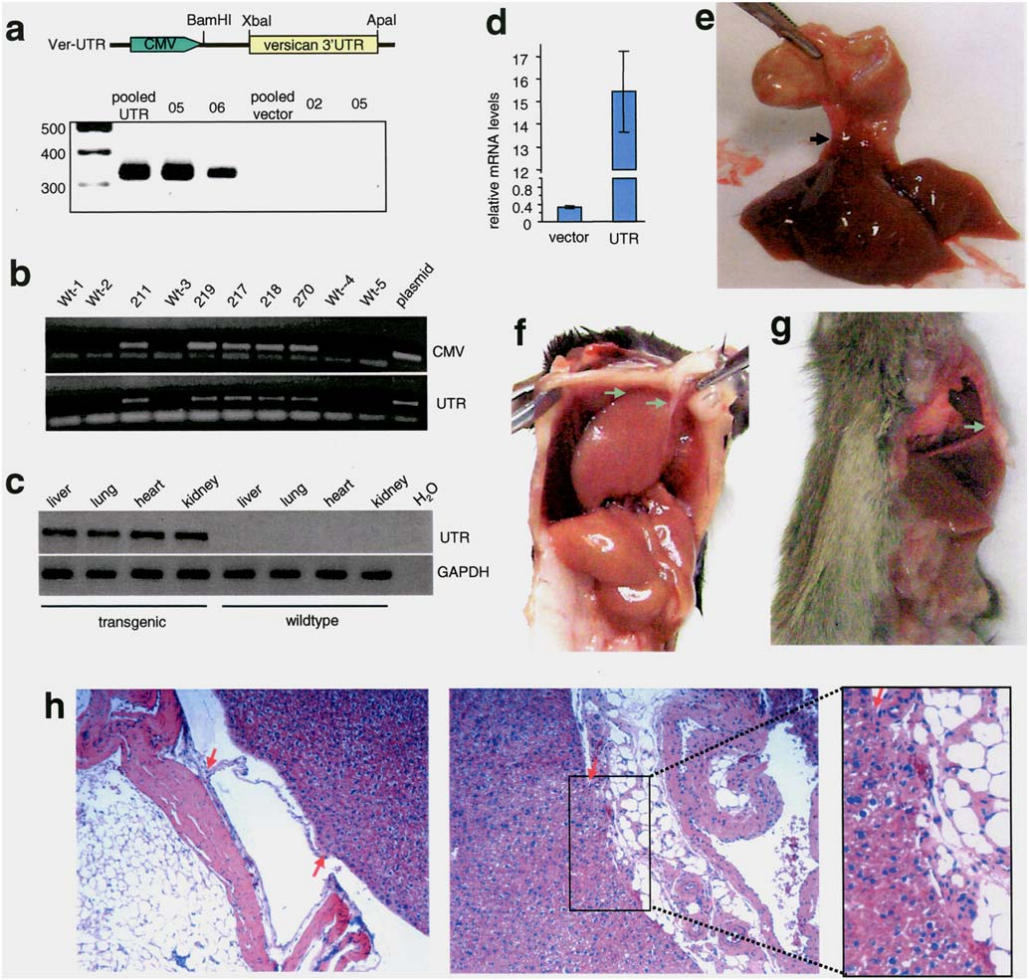
Organ adhesion detected in transgenic mice expressing versican 3′UTR. (a), A fragment of versican 3′UTR (700 bp) immediately after the stop codon containing a small fragment at the 3′ of versican coding sequence was inserted into the pcDNA3.1 plasmid, downstream of the CMV promoter, between XbaI and ApaI sites, producing Ver-UTR construct. Total RNA was prepared from one pooled cell line and two individual clones stably transfected with Ver3′UTR or the empty vector, subjected to RT-PCR, and analyzed on agarose gel electrophoresis. Expression of the 3′UTR was confirmed. (b) Genotyping PCR was performed on tail DNA extracted from the same litter of F1 using two pairs of primers amplifying the promoter region (CMV) and the downstream transcript versican 3′UTR. (c) Expression of the transgene was analyzed by RT-PCR using RNAs isolated from different organs of 3′UTR transgenic mice. (d) The levels of versican 3′UTR were analyzed by real-time PCR in Hek293 cells transfected with the 3′UTR construct or a control vector (detecting endogenous versican). The levels of 3′UTR in the 3′UTR-transfected cells were over 45-fold that of the control. (e) Photograph showing adhesion of the liver to the stomach. (f) Photograph showing organ adhesion occurred between liver and diaphragm. One piece of liver was adhered to the diaphragm. (g) A liver was adhered to the internal body walls of the mouse. (h) Paraffin sections of the adhesion tissues were stained with hematoxylin and eosin (H&E). Both photographs show the linking between the livers and connective tissues.

To study the effect of versican 3′UTR on modulating miRNA functions, we cloned and placed the 3′UTR under the control of the CMV promoter. The 3′UTR was transcribed as expected (Fig 1a). The fragment containing the transcription unit was cropped for the generation of transgenic mice and we obtained four versican 3′UTR transgenic founder animals. All were bred with wildtype mice and we obtained a total of twelve litters of F1 mice. Genotyping was performed by PCR using two different pairs of primers and the result from one litter is shown in Fig 1b. Expression of the transgene in a number of organs was confirmed by RT-PCR (Fig 1c). Real-time PCR analysis indicated that the levels of the 3′UTR were 45-fold higher than that of wild-type (Fig 1d). A number of the positive F1 and wild-type mice were sacrificed and examined. We observed that in some 3′UTR mice, the livers were strongly adhered to the stomach (Fig 1e). In some others, the livers adhered with connective tissues sticking to the internal body wall (Fig 1f, arrows) or directly adhered to it (Fig 1g). In a different transgenic line, stomach adhesion (Fig S1b) and liver adhesion (Fig S1c) were also observed. These adhering organs were subjected to histological analysis by hematoxylin and eosin (H&E) staining. Adhesion of livers with connective tissues could be clearly seen (Fig 1h, arrows).

### Expression of versican 3′UTR induces cell adhesion

**Figure 2:**
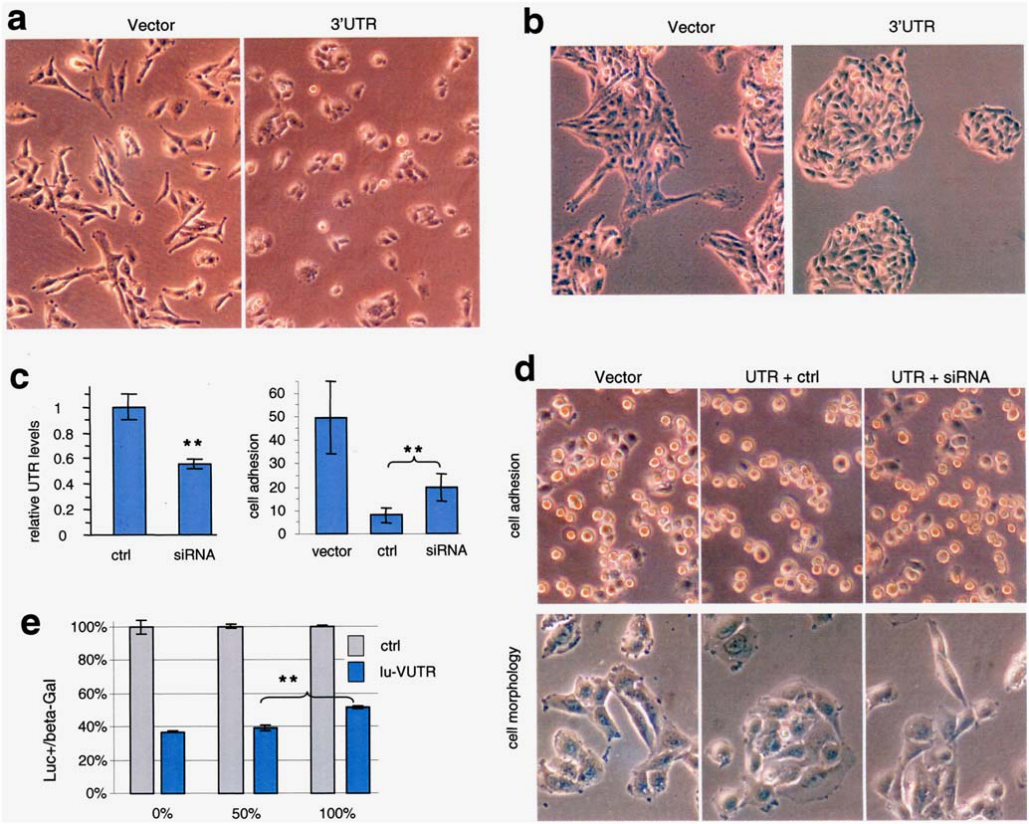
Cell adhesion and morphology affected by versican 3′UTR. Vector- or 3′UTR-transfected cells were inoculated in tissue culture dishes overnight. Cell morphology was examined under a light microscope (a). Two days after cell inoculation, the 3′UTR-expressing cells exhibiting island-like morphology (b). The 3′UTR-transfected cells were transiently transfected with siRNA targeting the 3′UTR or a control sequence, followed by real-time PCR analysis of the 3′UTR levels (c, left). Cell adhesion of the two groups of cells was compared with the vector-transfected cells. Down-regulation of the 3′UTR levels increased cell adhesion (c, right). Typical results of cell adhesion are shown (d, left). Cell morphology was also examined. Down-regulation of the 3′UTR levels reversed the morphology (d, right). Luciferase reporter vector harboring the versican 3′UTR was co-transfected with the versican 3′UTR construct at different amount combined with a control vector in U343 cells. Increase rations of versican 3′UTR bound more endogenous miR199a* and thus freeing the translation of luciferase protein, resulting in higher levels of luciferase activities (e).

To study how versican 3′UTR affected organ adhesion, we examined its effects on cell lines stably expressing the 3′UTR. Human astrocytoma cell line U343 was stably transfected with the 3′UTR construct or an empty vector. The cells were cultured in DMEM containing 10% FBS at a cell density of 5×10^4^ cells/ml and examined under a light microscope and photographed. The 3′UTR-transfected cells attached to tissue culture plates slower than the vector-transfected cells (Fig S1d). After cell attachment, the UTR-transfected cells tended to adhere together and appeared less elongated (Fig 2a). Two days after cell inoculation, large groups of the 3′UTR-transfected cells could be detected, forming an island-like morphology (Fig 2b).

To confirm the direct effect of the 3′UTR, 4 different siRNAs complementary to the 3′UTR were synthesized. Down regulation of the 3′UTR was confirmed by real-time PCR amplifying a fragment of the 3′UTR (Fig 2c, left). Cell adhesion assays showed that transfection with the siRNAs partially rescued the reduced adhesion in the 3′UTR-transfected cells (Fig 2c, right). Furthermore, transfection of the siRNA also reversed the altered cell morphology (Fig 2d).

We developed a number of experiments to analyze the effects of the 3′UTR. It was linked with the construct expressing versican G3 domain [54], producing the G3-UTR. Cell lysates were prepared from U343 cells stably transfected with the G3 and G3-UTR constructs and were subjected to Western blot analysis probed with anti-G3 and anti-actin antibodies simultaneously. While actin levels were similar, G3 levels were much lower in cells transfected with the G3-UTR construct (Fig S2a). This result suggests that some endogenous miRNAs targeted the 3′UTR and repressed G3 expression.

The 3′UTR was also linked with the GFP expression unit (Fig S2b, upper). Fluorescent levels of U87 and U343 cells stably transfected with the GFP and GFP-UTR constructs were measured with flow cytometry. Cells transfected with the GFP-UTR construct produced lower levels of fluorescence than that transfected with the GFP construct (Fig S2b, middle and lower). Cells transfected with the GFP and GFP-UTR constructs were also examined under a light and fluorescent microscope. Transfection with the GFP-UTR construct produced lower levels of GFP than the controls (Fig S2c).

Furthermore, the 3′UTR was linked to the luciferase report vector producing the lu-VUTR construct. Cells that were transfected with different concentrations of lu-VUTR always produced lower levels of luciferase activity than cells transfected with a control construct (Fig S3a), suggesting repression of luciferase expression by endogenous miRNAs. Nevertheless, with increased concentrations of lu-VUTR, the relative luciferase activities increased when normalized with the control construct. This suggests that higher concentrations of lu-VUTR would absorb some endogenous miRNAs and free more lu-VUTR to be expressed. To confirm this, the lu-VUTR construct was co-transfected with the 3′UTR construct. Increased 3′UTR concentrations generated higher levels of luciferase activities in U343 cells (Fig 2e) and U87 cells (Fig S3b). These results suggest that versican 3′UTR plays a key role in the induction of adhesion.

### Versican 3′UTR interacts with miRNS-199a*

**Figure 3:**
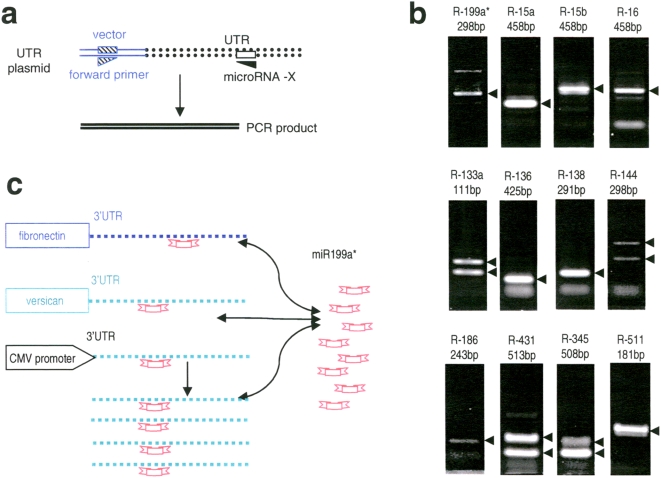
Targeting of versican 3′UTR with miRNAs. (a) Scheme for PCR method to test the interaction of miRNAs with versican 3′UTR. An oligonucleotide corresponding to miRNA-X is used as a reverse primer. It binds to the potential targeting sites on the antisense strand of the 3′UTR construct, depending on the extent of complementation. One forward primer docked on a different location of the vector was used to pair with the miRNA primer for PCR. (b) PCR products were obtained showing different size of products corresponding to the forward primer and the miRNA sequences. The expected sizes of PCR products are indicated with arrows. The miRNA sequences used were listed in Supporting Fig S1a. (c) Computational analysis showed that miR-199a* potentially targeted both versican and fibronectin 3′UTRs. Overexpression of versican 3′UTR would attract endogenous miR-199a* freeing versican mRNA and fibronectin mRNA for translation.

To test the direct interaction of miRNAs with the 3′UTR, we developed a PCR assay to test the potential binding interactions of the miRNAs with the 3′UTR and to validate the target sites *in silico*. This method assumes that the miRNAs targeting the 3′UTR could serve as a PCR-primer, and the PCR products could be generated by a 5′UTR-specific primer pairing with the miRNA primer. The nucleotide sequence of miRNA-X primer corresponds to the RNA sequence of miRNA-X but with the substitution of uridine for thymidine (Supporting Fig S1a). The versican 3′UTR construct was used as a PCR-template (Fig 3a).

We analyzed the potential miRNAs that bind to the 3′UTR of the Ver-UTR construct. A number of candidates with low binding energy were detected. We tested 17 different miRNAs selected from the potential candidates for the 3′UTR of versican. As shown in Fig 3b and Supporting Fig S3c, PCR products with expected sizes were generated by using two different annealing temperatures. There was no correlation between positive PCR results and the G/C content or the melting temperature of primers, which indicated the specificity of miRNAs toward the 3′UTR of versican. This method can be used to screen miRNAs that bind to the 3′UTR of interest. It is more efficient than the commonly-used luciferase assay and can be used to confirm the candidate binding sites as identified by other tools. Nevertheless, this is not to replace any existing methods. Rather, it adds an alternative approach to identify miRNAs targeting a 3′UTR of interest. It also produces a shorter list of candidates for validation by luciferase activity assays and transfection experiments.

It is expected that one 3′UTR contains many miRNA binding sites and one miRNA might target many 3′UTRs. This would create a balanced network composing of the synergy and counteraction of miRNA-3′UTR interactions. The homeostatsis of miRNAs and miRNA-binding sites might be disrupted through changes in the expression of transcripts or miRNAs. Over-expression of the versican 3′UTR would affect the levels of free miRNAs through binding the miRNAs, which normally regulate versican expression by targeting its 3′UTRs. The formation of miRNA-3′UTR transcript duplex thus decreases the functional miRNA levels. This interaction would affect protein expression, leading to the functional consequences.

Using the computation algorithm FindTar (http://bio.sz.tsinghua.edu.cn/findtar), we found a great number of miRNAs potentially targeting the versican 3′UTR. One of the positive miRNAs that showed interaction with the versican 3′UTR is miR-199a*. Using the online search engine TargetScan4.0 (www.targetscan.org), we found a great number of genes that are potentially targets of miR-199a* including versican (Genbank access number NM_004385) and fibronectin variant 1-6 (Genbank access number NM_212482, NM_212475, NM_002026, NM_212478, NM_212476, and NM_212474). Fibronectin is an extracellular glycoprotein which binds to integrins and mediates cell adhesion, proliferation, tissue development, and life maintenance [55], [56]. It helps maintain cell shape by lining up cells and organizing their intracellular cytoskeleton. Our previous studies indicated that versican regulates cell proliferation, adhesion, aggregation, blood coagulation, and angiogenesis [57]–[62]. Thus, we hypothesized that the overexpression of versican 3′UTR would attract endogenous miR-199a*, freeing versican and fibronectin mRNA for translation. Increased versican expression would produce the consequences observed in this study.

### Transgenic expression of versican 3′UTR increases levels of endogenous versican

**Figure 4:**
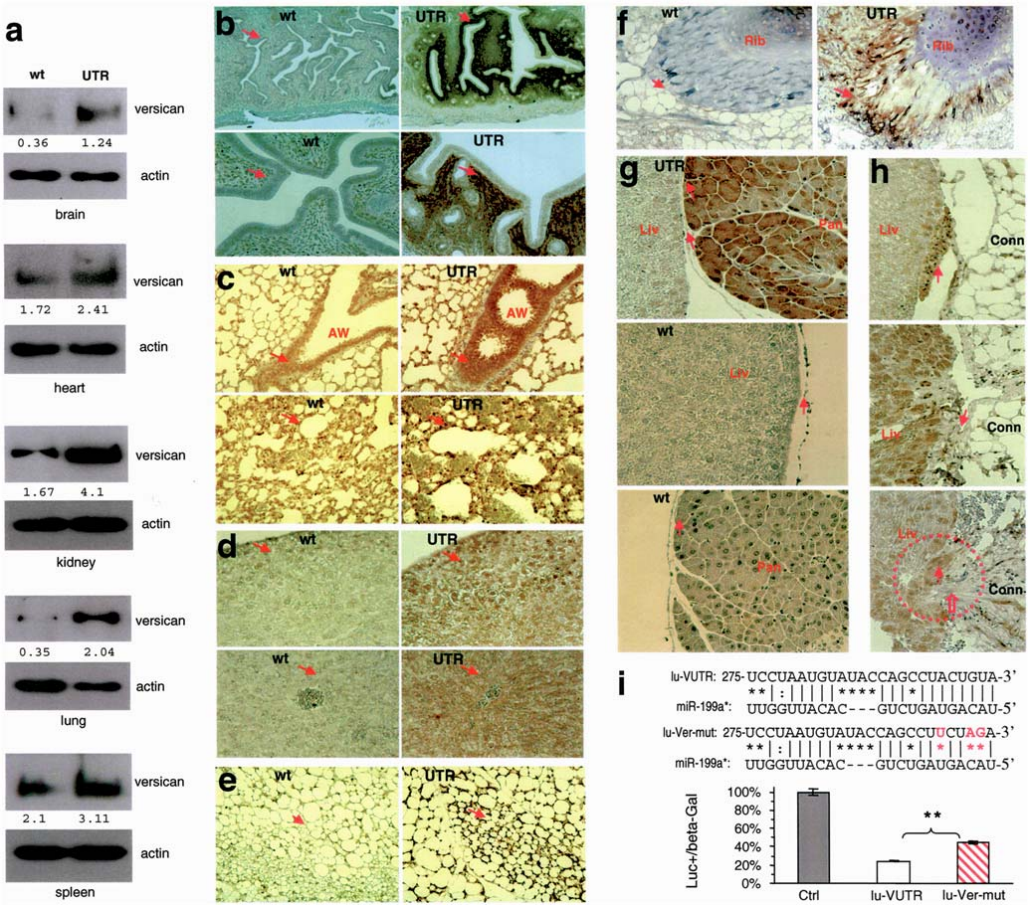
Up-regulation of versican expression in transgenic mice expressing versican 3′UTR. (a) Protein lysates were prepared from different organs and subjected to western blot analysis probed with anti-versican antibody. Detection of β-actin on the same membranes served as a loading control. Increased versican expression was detected in the organs harvested from the transgenic mice. (b–f) Paraffin sections of tissues from reproductive system (b), lung (c), liver (d), connective tissues (e), and rib (f) of the 3′UTR-transgenic and wild-type mice were stained with anti-versican antibody. In all sections shown, versican expression levels in the transgenic mice were higher as compared with the wild-type (arrows). (g) The levels of versican expression were higher in the pancreas that adhered to the liver (arrows). (h) The levels of versican expression were higher in the junctions between liver and the surrounding connective tissues (arrows). In the lower panel identified by the circle, some liver tissues (solid arrow) and connective tissues (open arrow), stained with anti-versican antibody, were mixed up, and no border could be identified between the different tissues. (i) Versican 3′UTR (nucleotides 275–299 of the 3′UTR, Upper) was found to be the potential target of miR-199a*. A versican 3′UTR was cloned and inserted into the luciferase reporter vector pMir-Report. Mutations were generated on the potential target sequence (red color). Lower, U343 cells were co-transfected with the miR-199a construct and the luciferase reporter construct harboring the versican 3′UTR (lu-VUTR) or the mutant construct (lu-Ver-mut). As a negative control, the luciferase reporter construct was engineered with a non-related fragment of cDNA (Ctrl). Luciferase activity assays indicated that the miR-199a construct repressed luciferase activities when it harbored the versican 3′UTR, which was abolished when the potential miR-199a* target site was mutated. Significant differences are indicated by asterisks. ** Error bars, SEM (n = 3), ** p<0.01.

To test whether the expression of versican 3′UTR affected versican expression, we prepared protein lysates from the brain, heart, kidneys, lungs, and spleen and analyzed versican expression by western blotting. Our experiments showed that the 3′UTR transgenic mice expressed higher levels of versican compared with the wild-type mice (Fig 4a). The organs were also subjected to histological analysis probed with anti-versican antibody. In the reproductive system, versican levels were much higher in the 3′UTR mice as compared with the wild-type mice (Fig 4b, arrows). As well, versican levels were much higher in the airways and pulmonary blood vessels of the 3′UTR lung (Fig 4c, arrows), in the livers (Fig 4d), the connective tissues (Fig 4e), and the ribs of the 3′UTR mice compared with the wild-type organs (Fig 4f). In the adhering organs of livers and pancreas, versican expression in the 3′UTR pancreas was much higher compared with the wild-type (Fig 4g). Normally, the liver does not adhere to any organ. However, in the 3′UTR mice, the liver adhered tightly to others such as pancreases and connective tissues of the body wall (Fig 4h). In the extreme case, the liver lost its smooth edges (Fig 4h, middle) and even merged with the connective tissue (Fig 4h, bottom). Increased versican expression was also detected in the junction areas of stomach and connective tissues (Fig S3d). The adhesion tissues were also immunostained for type I collagen expression, as type I collagen is an important adhesion molecule [63], [64]. We found that the junction of adhesion tissues expressed high levels of type I collagen (Fig S3e). This result indicated that type I collagen plays a role in versican 3′UTR-induced organ adhesion.

To confirm if versican was a target of miR-199a*, U343 cells were co-transfected with versican 3′UTR-luciferase construct (lu-VUTR) or the mutant lu-Ver-mut, in which the miR-199a* target site was mutated by nucleotide replacement (Fig 4i, upper). Luciferase activity assays showed that while presence of the versican 3′UTR reduced luciferase activities, mutation of the miR-199a* target site partially rescued luciferase activities (Fig 4i, lower). This suggests that miR-199a* repressed versican expression by targeting the 3′UTR. Furthermore, incomplete rescue of luciferase activities suggests that the versican 3′UTR is also targeted by other endogenous miRNAs.

### Transgenic expression of versican 3′UTR promotes fibronectin expression

**Figure 5:**
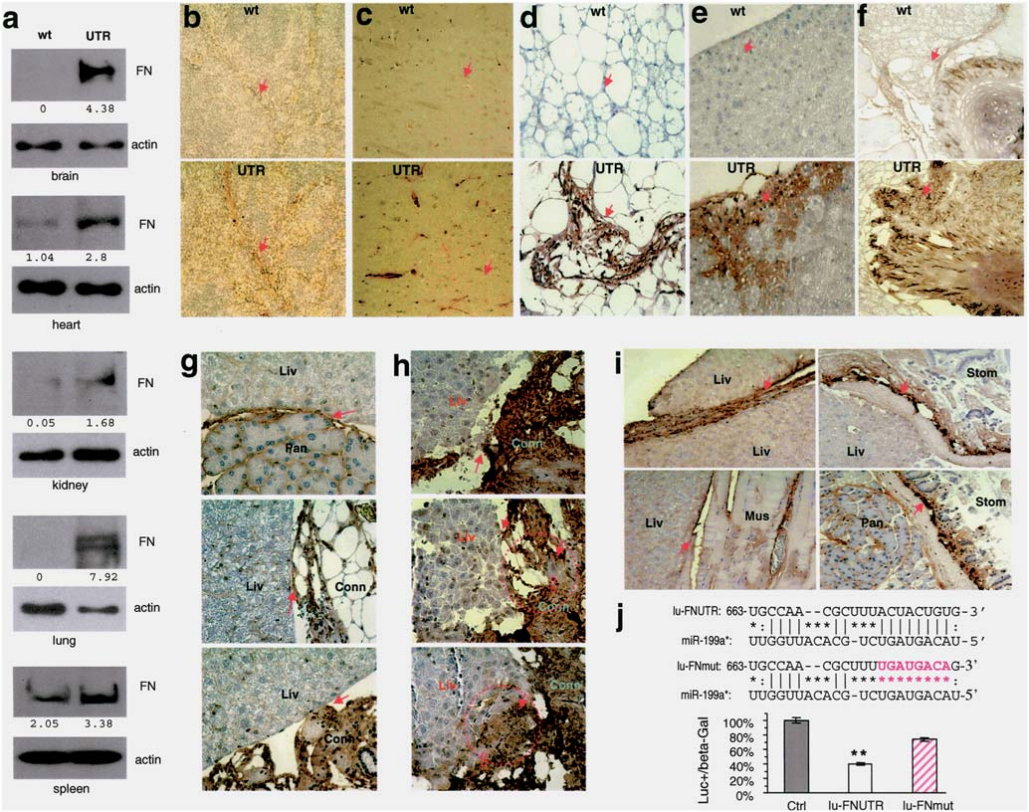
Up-regulation of fibronectin expression in transgenic mice expressing versican 3′UTR. (a) Protein lysates were prepared from different organs and subjected to western blot analysis probed with anti-fibronectin antibody. Detection of β-actin on the same membranes served as a loading control. Increased fibronectin expression was detected in the organs harvested from the transgenic mice. (b–f) Paraffin sections of tissues from spleen (b), brain (c), connective tissue (d), liver (e), and rib (f) of the 3′UTR-transgenic and wild-type mice were stained with anti-fibronectin antibody. In the transgenic spleen, the connective tissue structures expressed higher levels of fibronectin than the wild-type tissues did (arrows). In the transgenic brain, fibronectin expression was higher in the blood vessels (arrows). In the connective tissues of the transgenic mice, some areas expressed higher levels of fibronectin as compared with the wild-type (arrows). In the transgenic liver, fibronectin expression was higher along the edges of the liver than the wild-type liver. (g) The levels of fibronectin expression were higher in the 3′UTR pancreas that adhered to the liver (arrows). The connective tissues that adhered to the livers also expressed high levels of fibronectin. (h) In a different mouse, the 3′UTR connective tissues, while expressed high levels of fibronectin, were strongly adhered with the liver. Pulling out the connective tissue severely damaged the liver surface (upper panel, arrow). In the areas identified by the circles, some liver tissues (solid arrow) and connective tissues (open arrow, stained with anti-fibronectin antibody) were completely merged. (i) Adhesion of different organs was detected between liver/liver, liver/muscle, liver/stomach, and pancreas/stomach. The junctions between the organs expressed high levels of fibronectin (arrows). (j) Fibronectin 3′UTR (nucleotides 663–683 of the 3′UTR, Upper) was found to be the potential target of miR-199a*. A fibronectin 3′UTR was cloned and inserted into the luciferase reporter vector pMir-Report. Mutations were generated on the potential target sequence (pink color). Lower, U343 cells were co-transfected with the miR-199a construct and the luciferase reporter construct harboring the fibronectin 3′UTR (lu-FNUTR) or the mutant construct (lu-FNmut). Luciferase activity assays indicated that the miR-199a construct repressed luciferase activities when it harbored the fibronectin 3′UTR, which was abolished when the potential miR-199a* target site was mutated. Significant differences are indicated by asterisks. ** Error bars, SEM (n = 3), ** p<0.01.

To test whether the expression of versican 3′UTR affected fibronectin levels, we prepared protein lysates from the brain, heart, kidneys, lungs, and spleen and analyzed fibronectin expression by western blotting. Our experiments showed that the 3′UTR transgenic mice expressed higher levels of fibronectin compared with the wild-type mice (Fig 5a). The organs were also subjected to histological analysis probed with anti-fibronectin antibody. In the spleen, fibronectin levels were higher in the connective tissue structures of the 3′UTR mice as compared with the wild-type mice (Fig 5b, arrows). As well, fibronectin levels were higher in the brain (Fig 5c, arrows), connective tissues (Fig 5d), liver (Fig 5e), and ribs (Fig 5f) of the 3′UTR mice compared with the wild-type organs. In the adhering organs of livers and pancreas, fibronectin expression in the 3′UTR pancreas was clearly detected (Fig 5g, upper). In the adhesion organs associated with liver and connective tissue, fibronectin was strongly expressed in the connective tissues adhering to the liver (Fig 5g, middle and lower). In the extreme cases, where the liver lost its smooth edges and mixed up with the connective tissue, fibronectin translation was greatly promoted (Fig 5h). Furthermore, fibronectin levels were much higher in the adhesion areas between liver/liver, liver/stomach, liver/muscle, and pancreas/stomach (Fig 5i).

To confirm that fibronectin was a target of miR-199a*, we cloned the fragment of fibronectin 3′UTR containing the miR-199a* target site. The fragment was inserted into the luciferase reporter vector producing a construct lu-FNUTR. The potential target site of miR-199a* was mutated producing lu-FNmut. U343 cells were co-transfected with fibronectin 3′UTR-luciferase construct (lu-FNUTR) or the mutant lu-FNmut (Fig 5j, upper). Luciferase activity assays showed that insertion of the fibronectin 3′UTR repressed luciferase activities as compared with the control (Fig 5j, lower). Mutation of the miR-199a* target site partially rescued luciferase activities. This suggests that miR-199a* repressed fibronectin expression by targeting the 3′UTR of fibronectin. Incomplete rescue of luciferase activities suggests that the fibronectin 3′UTR is also targeted by other endogenous miRNAs. When lu-FNUTR was co-expressed with Ver-UTR, luciferase activities increased (Fig S4f), suggesting a competition of miRNAs between Ver-UTR and lu-FNUTR.

Our experiments indicated that the functions of miRNAs can be regulated by a fragment of non-coding transcript. Genomic deletion/truncation leading to translational silencing produces mutant phenotypes as the consequence of protein loss/mutation accompanied by the existence of non-coding/mutation transcripts. This strategy has been extensively used to knock-out genes of interest in studying gene functions. After gene knock-out, the protein is no longer expressed, but it is conceivable that the mutated genes are still able to produce non-coding transcripts. Sometimes, no detectable phenotypes are obtained, and it is said that the mutated/lost proteins may be compensated with others. Although compensation by other proteins is possible, the non-coding transcript may play an important role in compensation and balancing the mutant entity. Our results demonstrate a dramatic functional consequence by expressing a non-coding transcript. Exogenous expression of the 3′UTR construct altered the expression of some proteins functionally associated with the 3′UTR. It is possible that the 3′UTR can play more diverse roles than the protein expressed by the same transcript, although proteins are the executants of biological activities. Therefore, while analyzing the results of gene knock-out, one may need to consider the effects of not only the proteins but also the remaining non-coding transcripts. Our results that exogenous expression of the versican 3′UTR promoted versican expression suggest that each mRNA may exert at east two functional roles: through protein translation and miRNA regulation.

The human genome contains a large number of pseudogenes, which are nearly as abundant as the functional genes and therefore appear to be an important component in the genome. It has been reported that there are approximately 20,000 putative pseudogenes in the human genome [2]. A large number of pseudogenes are found to be transcribed. Analysis of chromosome 22 indicated that approximately 20% of the pseudogenes are potentially transcribed [3]. Pseudogene transcription has also been reported in other species including fly, mouse, cow, and chimpanzee [4]. The assumption that pseudogenes are dysfunctional is based on the fact that pseudogenes do not code for proteins. It is possible that these non-coding transcripts of the pseudogenes play important roles as modulators in miRNA functions.

It seemed that expression of the 3′UTR produced a similar functional role as the miRNA inhibitor. However, one 3′UTR has the capacity to modulate multiple miRNAs, while one miRNA inhibitor can only affect one miRNA. In this sense, a 3′UTR may be able to exert diverse biological activities by modulating multiple miRNA functions. As such, an animal gene, with long sequence of the 3′UTR, may have the capacity of exerting complex biological activities. Furthermore, a long fragment of 3′UTR may be more stable, while the miRNA inhibitor may be readily degraded. Thus, expression of a 3′UTR may have great advantage in modulating cell activities. In terms of stability and functionality, the 3′UTR may be better than normal mRNA, in that a mRNA has many tasks to carry out, while a 3′UTR may only exist for miRNA binding. In the former case, binding with multiple factors involved in protein synthesis and formation of secondary structures would decrease the accessibility for miRNAs. In the latter case, a simple 3′UTR fragment would be much more accessible for miRNA binding. This may explain why expression of a non-coding fragment could serve as a vigorous tool and produce potent effects *in vitro* and *in vivo*. Furthermore, the 3′UTR may free a group of miRNAs that modulate mRNAs with related biological functions. Changes in miRNA regulation may improve translation of the network of related proteins, providing immediate, effective, and lasting biological effects. One could imagine that the future applications will be benefited by the application of the 3′UTR transcript in gene therapy.

## Materials and Methods

### Construct generation

To study the effect of versican 3′UTR on cell activities, we have cloned the 3′UTR by RT-PCR using two primers Huver-UTRNXbaI and Huver-UTRCApaI. The PCR product was digested with restriction enzymes XbaI and ApaI and inserted into XbaI- and ApaI-opened pcDNA3.1 vector. After transformation, colony selection, DNA mini-preparation, and restriction enzyme digestion, the correct clones were sequenced to ensure identity of the 3′UTR.

A luciferase reporter vector (pMir-Report; Ambion) was used to generate the luciferase constructs. The 3′UTR of versican was cloned using 2 primers, HuverUTR-NSpeI and HuverUTR-CHindIII, by PCR. The PCR products were then digested with SpeI and HindIII and the fragment was inserted into a SpeI- and HindIII-digested pMir-Report Luciferase plasmid (Ambion), to obtain a luciferase construct, lu-VUTR. Primers used in this study are listed in the Supporting Fig S1a. A mutant construct was generated with two PCRs, one using two primers, HuverUTR-NSpeI and HuverUTR-mu-RXbaI, the other using HuverUTR-mu-FXbaI and HuverUTR-CHindIII. After restriction enzyme digestion, one with SpeI and XbaI, and the other with XbaI and HindIII, both fragments were ligated with pMir-Report vector opened with SpeI and HindIII.

A fragment of the 3′UTR of fibronectin was also cloned using 2 primers, FN1-N3′SacI and FN-199aC3′MluI, by RT-PCR. The PCR products were then digested with SacI and MluI and the fragment was inserted into a SacI- and MluI- digested pMir-Report Luciferase plasmid (Ambion), to obtain a luciferase construct, lu-FNUTR. A mutant construct was generated with two primers FN1-N3′SacI and FN-199aC3′MluI-Mut using similar approach.

To serve as a negative control, a non-related sequence was amplified from the coding sequence of the chicken versican G3 domain using 2 primers, chver10051SpeI and chver10350SacI. It is expected that there is no endogenous miRNA bind to this fragment as it is in the coding region. The PCR product was then inserted into a SpeI- and SacI-digested pMir-Report Luciferase plasmid.

### PCR identification for miRNA-UTR interaction

For *in vitro* binding of microRNAs with versican 3′UTR, a PCR method was developed. The pcDNA3.1 plasmid containing the 3′UTR of versican was used as the template in PCR. The forward primer located at the vector (pcDNA3.1hygro). The reverse primers for different miRNAs are listed in Supporting Fig S1a. In general, the PCR mixture (20 µl) contained: 100 mM KCl, 100 mM (NH_4_)_2_SO_4_, 200 mM Tris HCl, pH 8.75, 1.0% Triton X-100, 1 mg/ml BSA, 200 µM dNTPs, 2 µM forward primer, 2 µM reverse primer, 1 unit Taq DNA polymerase. The parameters for the PCR reaction were: one cycle at 95°C for 5 min; 35 cycles at 95°C for 1 min, 37°C for 1 min, 72°C for 1 min; and a final elongation step at 72°C for 10 min. The PCR products were then visualized with a 1.5% agarose gel stained with ethidium bromide.

### Generation and genotyping of the 3′UTR transgenic mice

The transgene was released from the plasmid by digestion with ApaLI and StuI. The digested product was fractionated by agarose gel electrophoresis and the 3 kb transgene fragment was excised from the gel, purified by Elutip mini-column (Schleicher and Schuell, Keene, NH) and then resuspended in injection buffer (10 mM Tris, pH 8.0 and 0.1 mM EDTA) at a concentration of 1 to 2 ng/µl. The transgene was microinjected into the male pronuclei of C57BL/6×CBA F_2_ mouse zygotes. Injected embryos were implanted into the oviducts of pseudopregnant recipient females using a standard protocol approved by the Animal Use Subcommittee of the University Council on Animal Care, The University of Western Ontario. Transgenic founder lines were maintained by backcrossing with C57BL/6×CBA F_1_ mice. Genotyping was performed by PCR, using primers EGFP-347F pairing with EGFP-668R (for CMV promoter and huver10861F pairing with Huversican-UTRCApaI (for versican 3′UTR), and tail snip or ear punch DNA as template. GAPDH served as a control using primers mo-Gapdh1F and mo-Gapdh250R. The transgenic mice were then transferred to Sunnybrook Research Institute (Toronto, Ontario). The methods for tissue harvest and analysis have been approved by the Animal Care Committee of Sunnybrook Research Institute, Ontario, Canada.

### Cell adhesion assay

Vector- or 3′UTR-transfected cells were plated onto culture dishes at a density of 4×10^5^ cells/ml and incubated for 30 min with DMEM containing 5% FBS. After 30 min, cells were fixed with 3.7% paraformaldehyde. Adhering cells were counted and cell images were captured using a phase-contrast microscope. Ten different fields (100×) were used for cell counting.

### Western blot

Organs were weighted and homogenized with lysis buffer containing protease inhibitors (150 mM NaCl, 25 mM Tris-HCl, pH 8.0, 0.5 M EDTA, 20% Triton X-100, 8 M Urea, and 1× protease inhibitor cocktail). Protein concentration was measured by Bio-Rad Protein Assay kit (#5000-0006). The lysates were subjected to SDS-PAGE and then transferred to nitrocellulose membranes probed with a primary antibody against versican (Lifespan Biosciences, LS-C25140), fibronectin (BD, 610078), or β-actin (Sigma-Aldrich) overnight at 4°C. After incubation with corresponding HRP-conjugated secondary antibodies, the membranes were washed, followed by detection with the ECL kit.

### Tissue slide preparation and immunohistochemistry

Organs were freshly excised and fixed in formalin overnight, immersed in 70% ethanol, embedded in paraffin, and sectioned by a microtome (Leica RM2255). The sections were de-paraffinized with xylene and ethanol and then boiled in a pressure cooker. After washing with Tris-Buffered-Saline (TBS) containing 0.025% Triton X-100, the sections were blocked with 10% goat serum and incubated with primary antibody against versican, fibronectin, or collagen Iα1 (Santa Cruz, sc-25974) in TBS containing 1% bovine serum albumin (BSA) overnight. The sections were washed and labeled with biotinylated secondary antibody, followed by avidin conjugated horse-radish peroxidase provided by the Vectastain ABC kit (Vector, PK-4000). The staining was developed by DAB kit (Vector, SK-4100). The slides were subsequently stained with Mayer's Hematoxylin for counter staining followed by slide mounting.

### Luciferase assay

U343 cells were seeded onto 24-well tissue culture plates at a density of 3×10^4^ cells/well in DMEM containing 10% FBS and maintained at 37°C for 24 hrs following the methods described by us recently [65], [66]. The cells were co-transfected with the luciferase reporter constructs and the 3′UTR construct by using Lipofectamine 2000. The cells were then collected by trypsin and lysed with a luciferase specific lysis buffer from Luciferase Assay Kit (Promega). Cells were centrifuged at 3000 rpm for 5 min. The supernatants were transferred into a black 96-well plate (3×10 µl) for luciferase activity measurement and into a transparent 96-well plate (3×50 µl) for β-gal activity determination. For the luciferase activity measurement, 70 µl of luciferase assay reagent was added into each well and the luciferase activity was detected by using microplate scintillation and luminescence counter (Packard, Perkin Elmer). For the internal control of β-gal activities, 90 µl of assay reagent (4 mg/ml ONPG, 0.5 M MgSO_4_, β-mercaptoethanol and 0.4 M sodium phosphate buffer) were added into each well. The plate was then incubated at 37°C for 60 min. The absorbance at 410 nm was measured by using a microplate reader (Bio-Tek Instruments, Inc.).

### Statistical Analysis

The results (mean values±SD) of all the experiments were subjected to statistical analysis by *t*-test. The level of significance was set at p<0.05.

## Supporting Information

Figure S1 (a) Primers used in this study. (b–c) Photographs showing organ adhesion occurred between liver and stomach (b), between liver and body (c) in a different transgenic line of mice. (d) Vector- or the 3′UTR-transfected cells were inoculated in tissue culture dishes for 2.5 hours. Cell adhesion was examined under a light microscope and photographed.
(8.01 MB TIF)

Figure S2 a, Upper, to test the effect of the versican 3′UTR, the versican G3 domain was linked with or without the 3′UTR producing G3 and G3-UTR constructs. Lower, cell lysates prepared from U343 cells stably transfected with the G3 and G3-UTR constructs were subjected to Western blot analysis probed with anti-G3 and anti-actin antibodies simultaneously. While actin levels were similar, G3 levels were much lower in cells transfected with the G3-UTR construct. Fig S2b, the GFP coding sequence was linked with or without the 3′UTR producing GFP and GFP-UTR constructs (Upper). Cells transfected with the GFP-UTR construct produced lower levels of GFP activities than that transfected with the GFP construct. The levels of fluorescent cells were quantified (Middle). Typical fluorescent levels of U87 and U343 cells transiently transfected with the GFP and GFP-UTR constructs were shown (Lower). Fig S2c, Cells transfected with the GFP and GFP-UTR constructs were also examined under a light and fluorescent microscope. Typical results are shown.
(8.51 MB TIF)

Figure S3 (a) U343 cells were transiently transfected with luciferase reporter vector harboring the versican 3′UTR (lu-VUTR) or a control sequence (ctrl). Luciferase activities were normalized using the control as 100%. The luciferase activities of lu-VUTR never reached the levels of the control, suggesting endogenous miRNAs targeting the versican 3′UTR. Nevertheless, the activities increased with higher does of plasmids, suggesting that increased supplies of versican 3′UTR absorbed some endogenous miRNAs freeing luciferase translation. (b) Luciferase reporter vector harboring the versican 3′UTR was co-transfected with the versican 3′UTR construct at different amount combined with a control vector in U87 cells. Increase amounts of versican 3′UTR bound more endogenous miR199a* and freeing the translation of luciferase protein, resulting in higher levels of luciferase activities. (c) PCR was performed using one forward primer docked on the vector and one of the mature miRNAs as indicated at a different temperature (35°C). PCR products were obtained showing different sizes of products corresponding to the forward primer and the miRNA sequences. (d) Photographs showing organ adhesion occurred between stomach and connective tissues. The sections were immunostained with anti-versican antibody showing that versican was deposited in the adhesion junction areas. (e) The adhesion tissues were sectioned and immunostainined with anti-type I collagen that normally deposits in wound healing areas. Collagen was expressed at high levels in the areas of tissue adhesion.
(9.01 MB TIF)

Figure S4 Paraffin sections of adhesion organs from a different transgenic line of mice were stained with anti-fibronectin antibody. The levels of fibronectin expression were higher in the adhesion junctions between liver and pancreas (a), between liver and connective tissue (b–e), and between liver and liver (e, right). Luciferase reporter vector harboring the fibronectin 3′UTR was co-transfected with the versican 3′UTR construct at different amount combined with a control vector in U343 cells. Increased ratios of versican 3′UTR bound more endogenous miR199a* and thus freeing the translation of luciferase protein, resulting in higher levels of luciferase activities (f).
(9.31 MB TIF)
