# Supplementary figures and images for: A 3′-Untranslated Region (3′UTR) Induces Organ Adhesion by Regulating miR-199a* Functions
Source: PLoS One. 2009 Feb 18;4(2):e4527. doi: 10.1371/journal.pone.0004527 (PMC2638016; doi:10.1371/journal.pone.0004527)

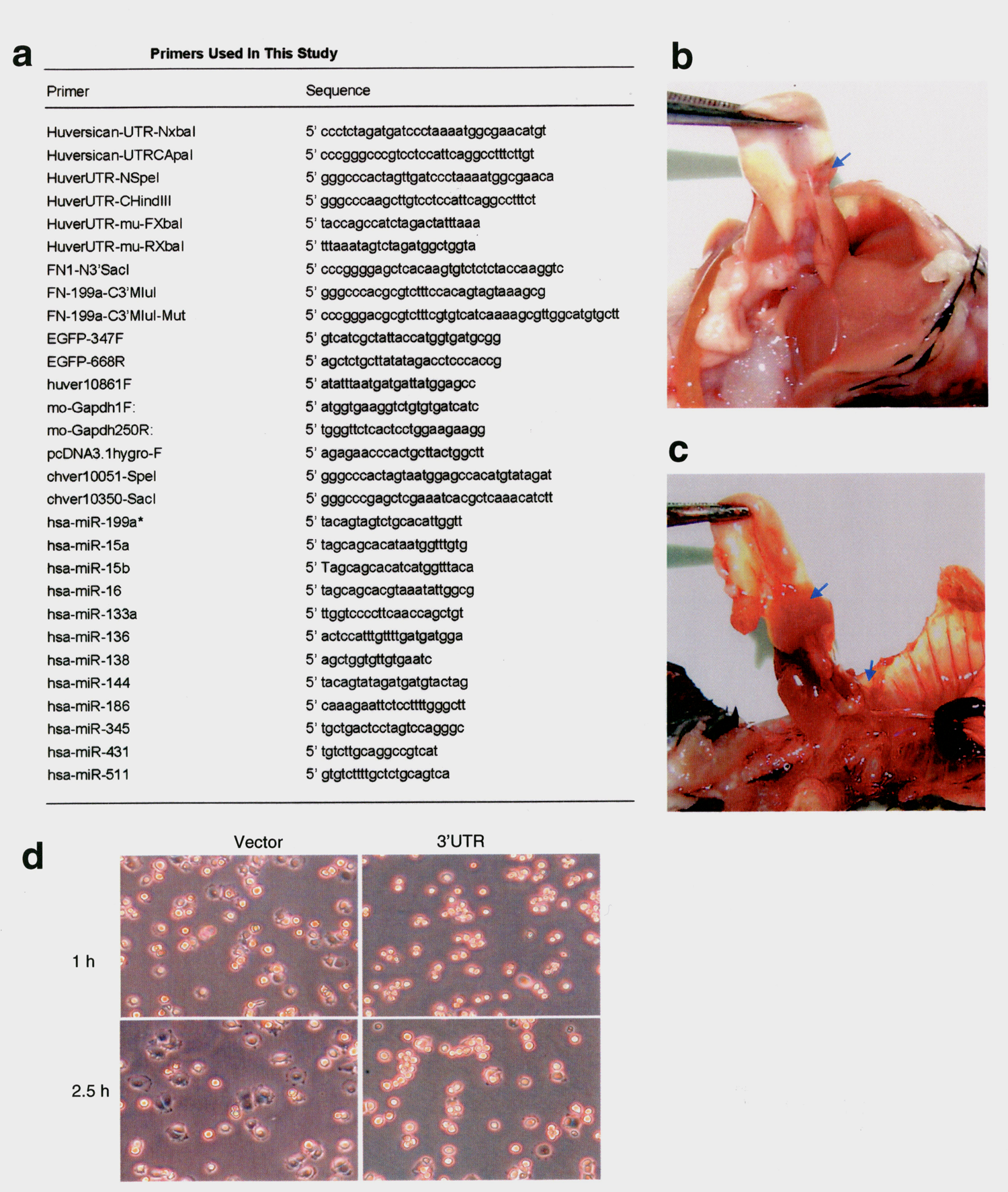

Supplement: Figure S1 — (a) Primers used in this study. (b–c) Photographs showing organ adhesion occurred between liver and stomach (b), between liver and body (c) in a different transgenic line of mice. (d) Vector- or the 3′UTR-transfected cells were inoculated in tissue culture dishes for 2.5 hours. Cell adhesion was examined under a light microscope and photographed. (8.01 MB TIF) [file pone.0004527.s001.tif]

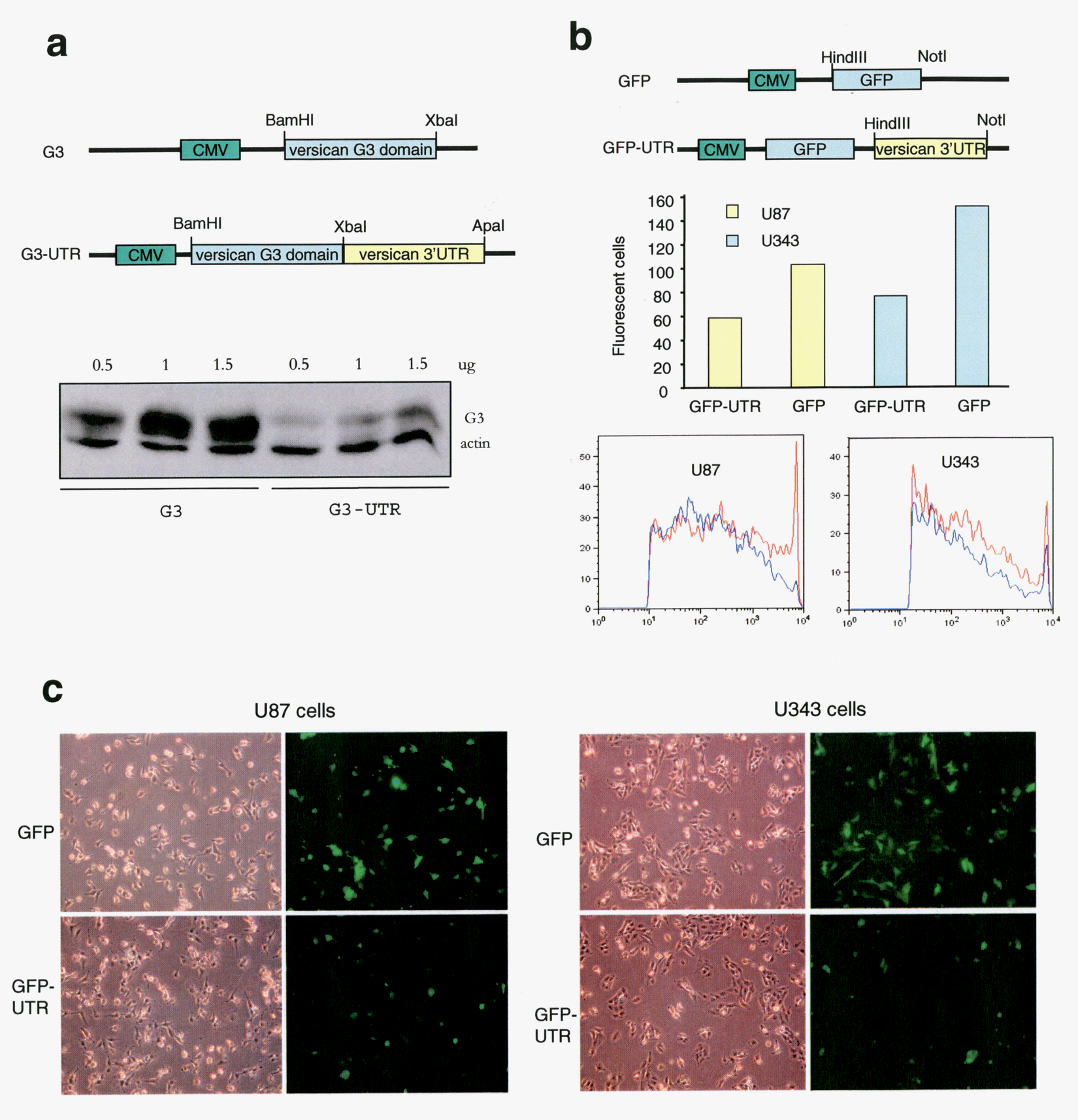

Supplement: Figure S2 — a, Upper, to test the effect of the versican 3′UTR, the versican G3 domain was linked with or without the 3′UTR producing G3 and G3-UTR constructs. Lower, cell lysates prepared from U343 cells stably transfected with the G3 and G3-UTR constructs were subjected to Western blot analysis probed with anti-G3 and anti-actin antibodies simultaneously. While actin levels were similar, G3 levels were much lower in cells transfected with the G3-UTR construct. Fig S2b, the GFP coding sequence was linked with or without the 3′UTR producing GFP and GFP-UTR constructs (Upper). Cells transfected with the GFP-UTR construct produced lower levels of GFP activities than that transfected with the GFP construct. The levels of fluorescent cells were quantified (Middle). Typical fluorescent levels of U87 and U343 cells transiently transfected with the GFP and GFP-UTR constructs were shown (Lower). Fig S2c, Cells transfected with the GFP and GFP-UTR constructs were also examined under a light and fluorescent microscope. Typical results are shown. (8.51 MB TIF) [file pone.0004527.s002.tif]

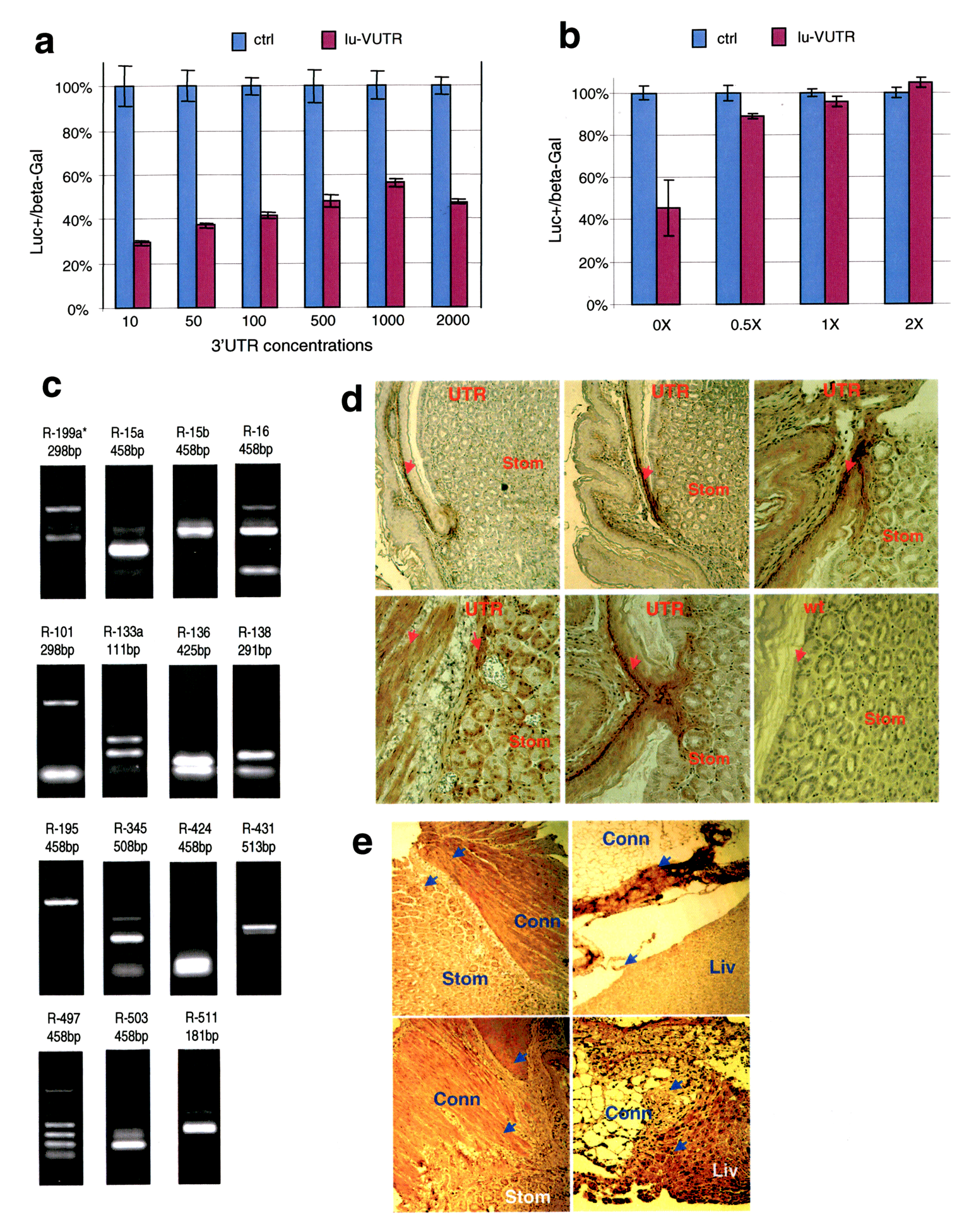

Supplement: Figure S3 — (a) U343 cells were transiently transfected with luciferase reporter vector harboring the versican 3′UTR (lu-VUTR) or a control sequence (ctrl). Luciferase activities were normalized using the control as 100%. The luciferase activities of lu-VUTR never reached the levels of the control, suggesting endogenous miRNAs targeting the versican 3′UTR. Nevertheless, the activities increased with higher does of plasmids, suggesting that increased supplies of versican 3′UTR absorbed some endogenous miRNAs freeing luciferase translation. (b) Luciferase reporter vector harboring the versican 3′UTR was co-transfected with the versican 3′UTR construct at different amount combined with a control vector in U87 cells. Increase amounts of versican 3′UTR bound more endogenous miR199a* and freeing the translation of luciferase protein, resulting in higher levels of luciferase activities. (c) PCR was performed using one forward primer docked on the vector and one of the mature miRNAs as indicated at a different temperature (35°C). PCR products were obtained showing different sizes of products corresponding to the forward primer and the miRNA sequences. (d) Photographs showing organ adhesion occurred between stomach and connective tissues. The sections were immunostained with anti-versican antibody showing that versican was deposited in the adhesion junction areas. (e) The adhesion tissues were sectioned and immunostainined with anti-type I collagen that normally deposits in wound healing areas. Collagen was expressed at high levels in the areas of tissue adhesion. (9.01 MB TIF) [file pone.0004527.s003.tif]

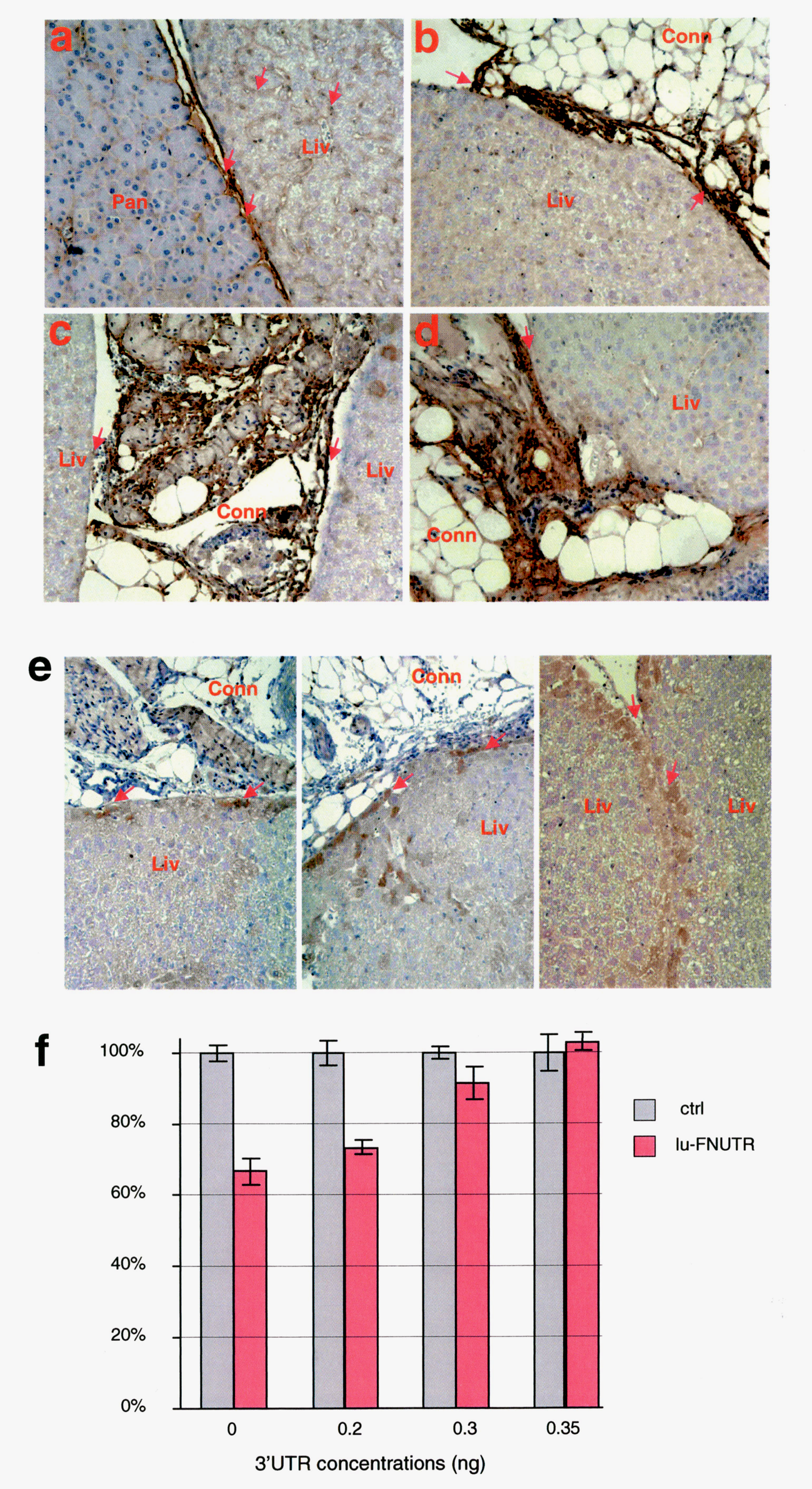

Supplement: Figure S4 — Paraffin sections of adhesion organs from a different transgenic line of mice were stained with anti-fibronectin antibody. The levels of fibronectin expression were higher in the adhesion junctions between liver and pancreas (a), between liver and connective tissue (b–e), and between liver and liver (e, right). Luciferase reporter vector harboring the fibronectin 3′UTR was co-transfected with the versican 3′UTR construct at different amount combined with a control vector in U343 cells. Increased ratios of versican 3′UTR bound more endogenous miR199a* and thus freeing the translation of luciferase protein, resulting in higher levels of luciferase activities (f). (9.31 MB TIF) [file pone.0004527.s004.tif]
